# Public perception of COVID-19 in Saudi Arabia during the Omicron wave: recommendations for policy improvement

**DOI:** 10.3389/fpubh.2025.1419891

**Published:** 2025-02-27

**Authors:** Mohammed J. Almalki, Amani A. Alotaibi, Abdulrahman M. Jabour, Ali Elamin, Wafa A Hetany, Hamza Abdullah M. Adam, Hassan N Moafa, Ahmed S. Alamer, Anwar Alsharif, Sami M. Almalki

**Affiliations:** ^1^Department of Public Health, College of Nursing and Health Sciences, Jazan University, Jazan, Saudi Arabia; ^2^Jazan University Hospital, Jazan University, Jazan, Saudi Arabia; ^3^General Directorate of Prisons Health, Ministry of Interior, Jazan, Saudi Arabia

**Keywords:** COVID-19, pandemics, public health, public perceptions, policy, Saudi Arabia

## Abstract

**Background:**

The emergence of new SARS-CoV-2 variants makes it difficult to forecast potential epidemiological changes. This study investigates Saudi citizens’ perceptions of COVID-19 during the Omicron wave.

**Methods:**

We conducted a cross-sectional study using an online survey and a convenience sample of 746 participants. The survey included questions about demographics, anxiety levels, and perception of COVID-19 during the Omicron wave.

**Results:**

Our findings revealed that 27.3% of the participants believed that COVID-19 cases would decrease, while 30.2% believed that cases would increase; the remaining 42.5% were uncertain. When asked about the primary reasons for expecting a rise in COVID-19 cases, the two most frequently cited causes were non-adherence to prevention measures (74.7%) and the high transmissibility of the virus (66.7%). Conversely, when asked about the primary reasons for expecting a decrease in COVID-19 cases, participants cited the availability of free vaccines (60.3%), government measures (59.9%), compliance with preventive measures (57.4%), and health awareness programs (44.1%). Multivariate logistic regression analysis indicated that anxiety about COVID-19 (AOR = 1.23, 95% CI: 1.15–1.32) and education level (AOR = 1.58, 95% CI: 1.11–2.25) were significant predictors of respondents’ expectations of increases or decreases in COVID-19 cases (*p* < 0.05). Around 46.2% of participants were moderately to highly worried about the reinstatement of lockdowns, while 36.2% reported moderate to high levels of anxiety related to COVID-19. Ordinal logistic regression analysis showed that respondents who reported higher levels of worry about the reinstatement of lockdowns were 1.28 times more likely to experience higher levels of anxiety related to COVID-19 (*p* < 0.05). A few participants were hesitant to adhere to preventive measures because they had already been vaccinated or believed that COVID-19 was not real or severe. This hesitancy raises public health concerns, suggesting that some individuals may underestimate the risks associated with COVID-19 and future pandemics.

**Conclusion:**

This study provides valuable insights into how Saudi citizens perceived COVID-19 during the Omicron wave. Understanding these perceptions can guide the development of public health policies, optimize resource allocation, help control the potential transmission of viral variants, and enhance preparedness for future pandemics.

## 1 Introduction

The coronavirus disease 2019 (COVID-19) pandemic has posed a global threat since its emergence in December 2019. More than 775 million people have been infected with the virus to date, with more than 7.7 million fatalities (1). The World Health Organization (WHO) has issued several statements about the ongoing COVID-19 outbreak, with more than 40,000 COVID-19 cases reported between May 13 and June 4, 2024 (2). The pandemic impacted many aspects of daily life, including the global economy, public services, education, healthcare, and social activities.

Saudi Arabia was one of the first countries in the Middle East to be affected by the COVID-19 pandemic. Since August 19, 2023, there have been 841,469 reported cases of COVID-19 in the country as well as more than 9,646 deaths (3). The Saudi government implemented several measures to contain the spread of the virus, including lockdowns: these involved the closure of schools, public offices, and businesses; restrictions on travel; limits on Hajj and Umrah activities; and controls on the usage of mosques for prayer (4–7). The government also launched a mass vaccination campaign. As of June 4, 2024, a total of 68.53 million doses of the COVID-19 vaccine had been administered, with 46% of the total population receiving at least one booster dose (8).

The emergence of new variants of severe acute respiratory syndrome coronavirus 2 (SARS-CoV-2), such as Omicron, along with its many lineages and new families, is an ongoing threat that requires collaborative partnerships to address (9, 10). For example, at least 30 SARS-CoV-2 genomes related to Omicron variants were reported in Saudi Arabia alone (11). The WHO raised the alarm about the Omicron variant on November 26, 2021, citing its high number of mutations and negative impact on COVID-19 epidemiology (12, 13) and highlighting its increased infectiousness compared to previous SARS-CoV-2 variants. The emergence of new variants makes it impossible to predict potential relevant epidemiological changes and it is possible that more hazardous variants could emerge in the future. These changes and uncertainties can affect people’s mental health, heighten anxiety, and affect perceptions of diseases and preventive measures.

A global cross-sectional online survey was distributed to assess people’s knowledge and perception of the Omicron variant of SARS-CoV-2 (14); the average knowledge score was found to be 3.18 out of 5, indicating that people had some knowledge of the variant though there was still room for improvement. In addition, perceptions of the fatality rate of Omicron varied from country to country (*p* = 0.05) (14). A study from the United States found that a majority of respondents were worried about the risk of contracting COVID-19 (15). However, the emergence of the Omicron variant led to an unexpected decline in negative perceptions and concerns among the general public while increasing them among individuals infected by COVID-19.

A cross-sectional study conducted in Jordan revealed a low average knowledge score about Omicron (4.19 ± 2.06 out of 8), suggesting that participants were relatively unaware of the variant (16). In addition, more than one-third of participants (38.6%) were unconcerned about the Omicron variant, indicating that a majority of respondents were worried or unsure about their perception of the disease. Another survey conducted in Iraq revealed that over half of the participants (56.5%) believed that Omicron was more contagious than previous variants (17), and the vast majority (86.5%) also believed that following the recommended protective measures would be ineffective.

In the Saudi context, a study was conducted in Riyadh during the Omicron wave to evaluate anxiety, depression, and fear in individuals with and without diabetes (18). The results revealed that, compared to non-diabetic participants, people with diabetes exhibited significantly higher levels of nervousness and anxiety (*p* = 0.001), depression and hopelessness (*p* = 0.004), and COVID-19-related fear (*p* = 0.037). Another study surveyed the experience of healthcare workers in Saudi Arabia regarding the Omicron variant (19); the results revealed that participants were more worried about the Omicron variant than the original, Alpha, and Delta strains (*p* < 0.001). Furthermore, uncertain individuals were more likely to experience stress (*r* = 0.326, *p* < 0.010). However, those with higher levels of resilient coping (*r* = −0.313, *p* < 0.010) as well as those who followed preventive measures and obtained information from the WHO website were better able to cope with stress. The negative perception of Omicron may also be attributed to its speedy transmission. An observational study at an eye care center in Riyadh found that 7.6% of ordinary patients tested positive for COVID-19 during the Omicron outbreak, although they were asymptomatic when swabbed (20).

This study aims to understand how Saudi citizens perceived COVID-19 during the Omicron wave as well as identify the factors that influenced these perceptions. Understanding these influences can guide responses to future potential epidemics, reduce the burden on public health, and inform relevant decisions. We examined the participants’ anxiety levels during the Omicron wave, their concerns about the possibility of another lockdown, their willingness to follow preventive measures, and whether they expected COVID-19 cases to increase or decrease. Evaluating concerns regarding potential future lockdowns creates an opportunity for adequate preparation, communication, and planning of necessary restrictions. Examining COVID-19-related anxiety also helps public health professionals to provide appropriate mental health support. Understanding people’s willingness to comply with preventive measures and their expectations regarding COVID-19 waves can help predict their responses to future waves as well as guide the planning of related health services. Finally, examining factors that influence people’s attitudes toward COVID-19 can assist in the development of targeted public health policies.

## 2 Materials and methods

### 2.1 Study design and sample

This study used a cross-sectional survey design. The minimum sample size of 385 was determined using Epi Info for Windows (21), taking into consideration the survey population (21.7 million) (22), margin of error (5%), confidence interval (95%), and response distribution (50%). All completed questionnaires were analyzed, resulting in a convenience sample of 746 participants.

### 2.2 Questionnaire

The initial draft of the study questionnaire was developed by the first author and validated by a group of experts, including two bilingual public health professors and co-authors of this article. The validation process included content and face validity (23). Upon approval, the questionnaire was translated into Arabic and validated by the same experts. The Arabic version was used for data collection, while the English version was presented for publication. Two bilingual public health professors and co-authors of this article reviewed both versions of the questionnaire to ensure coherence, clarity, and accuracy. The clarity of the Arabic version was also verified by a subgroup of the survey population.

The survey included questions on demographics, anxiety levels, perception of COVID-19 during the Omicron wave, and an open-ended section. Demographics queried included age, gender, marital status, care for dependents, education level, and whether the respondent had contracted COVID-19. The section on anxiety included two questions scored on a continuous scale from 1 to 10. These questions examined the level of anxiety among participants related to Omicron as well as their concerns about the possibility of another lockdown. A higher score on the scale indicates greater COVID-19-related anxiety or increased worry about the reinstatement of lockdowns. The percentile cut-off points (25th, 50th, and 75th percentiles) were used to categorize the level of anxiety experienced by respondents related to the Omicron wave and/or concerns about the reinstatement of lockdowns, resulting in three categories: low (1–3.33), moderate (3.34–6.67), and high (6.68–10). The section on perception included two questions graded on a five-point Likert scale about the participants’ willingness to follow preventive measures and whether they expected COVID-19 cases to increase or decrease. The responses ranged from “strongly disagree” to “strongly agree.” The third question in this section was a multiple-answer question about the different factors that influenced their perceptions. More details on the survey can be found in Supplementary material (24).

### 2.3 Data collection

Data were collected during February and March 2022 using an online survey that took an average of 4 min to complete. The researchers shared the survey link to their Twitter (X) followers and WhatsApp contacts, encouraging participants to continue sharing it across their social media networks. Eligibility criteria for participation included being Saudi Arabian, aged 18 years or older, and residing in Saudi Arabia during the pandemic.

This study was approved by the Research Ethics Committee of Jazan University in Saudi Arabia. Participants were given clear instructions about data confidentiality and the survey completion process. They were informed that their participation was voluntary and that they could withdraw at any time before submitting the survey. Participants were also asked to provide informed consent by answering a simple yes or no question.

### 2.4 Data analysis

The data were analyzed using the IBM SPSS statistics software, version 29. First, descriptive statistics were used to determine the characteristic demographics of the participants, whether they expected COVID-19 cases to increase or decrease during the Omicron wave, and the mean anxiety scores relating to Omicron and the reinstatement of lockdowns. Second, univariate and multivariate regression analyses were used to determine which factors were the strongest predictors of a respondent’s expectation regarding an increase or decrease in COVID-19 cases, including age, gender, education level, care for dependents, whether they had contracted COVID-19, adherence to the prevention measures, level of anxiety about Omicron, and worries about the reinstatement of lockdowns. Finally, an ordinal logistic regression model was used to assess the impact of the abovementioned independent variables on the participants’ anxiety about the Omicron variant. A *p*-value <0.05 indicates statistical significance.

## 3 Results

### 3.1 Demographic description

There were 774 responses to the survey. After 28 incomplete questionnaires were excluded, only 746 responses were included in the final sample. The average age of the participants was 29.18 years, with a standard deviation of 9.27 and a range of 18–55 years. Most participants were male (59.5%), unmarried (58.3%), and had completed university or higher education (64.9%). About 54.3% of respondents did not care for a dependent, while 63.3% had not contracted COVID-19. For more details, refer to Table 1.

**Table 1:** Participants characteristics.

Variables	*N*	%	Variables
Age	< 30 years	467	62.6
	30–40 years	169	22.7
	> 40 years	110	14.7
Gender	Female	302	40.5
	Male	444	59.5
Marital status	Unmarried	435	58.3
	Married	311	41.7
Education level	Pre-university	262	35.1
	University or higher	484	64.9
Care for dependents	No	405	54.3
	Yes	341	45.7
Contracted COVID-19	No	472	63.3
	Yes	274	36.7

### 3.2 Expectations regarding increases or decreases in COVID-19 cases during the omicron wave

**Figure 1:**
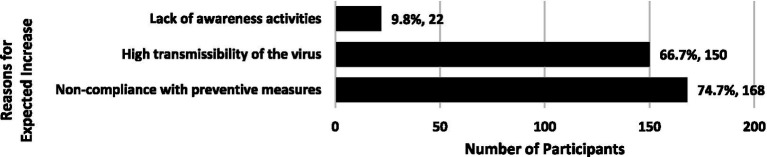
Primary reasons for expecting an increase in COVID-19 cases during the Omicron wave (*n* = 225). Percentage of those who expected an increase in COVID-19 cases. Participants were allowed to select multiple answers.

**Figure 2:**
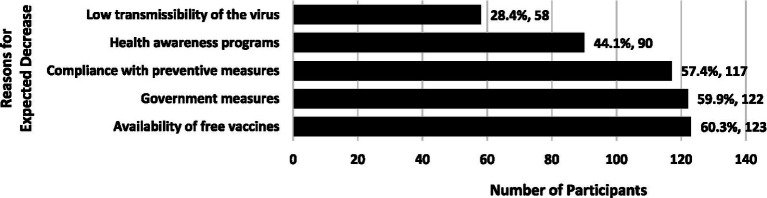
Primary reasons for expecting a decrease in COVID-19 cases during the Omicron wave (*n* = 204). Percentage of those who expected a decrease in COVID-19 cases. Participants were allowed to select multiple answers.

Less than one-third of the participants (*n* = 204; 27.3%) disagreed or strongly disagreed that COVID-19 cases would increase, while 30.2% (*n* = 225) agreed or strongly agreed that cases would increase; the remaining 42.5% (*n* = 317) were unsure about their expectations. Figures 1, 2 present the primary reasons provided for these expectations.

### 3.3 Intention to adhere to preventive measures

Although the vast majority of participants (*n* = 711; 95.3%) agreed or strongly agreed that they would voluntarily adhere to preventive measures against COVID-19, a small proportion (*n* = 8; 1%) disagreed or strongly disagreed, while 3.7% (*n* = 27) were hesitant. Several reasons were cited for rejecting adherence, including: “The disease is no longer as risky as it used to be,” “COVID-19 is not a real disease,” “COVID-19 is a flu-like disease that poses no real threat,” and “I am protected with three vaccine doses.” Table 2 provides a more detailed breakdown of the responses to this question.

**Table 2:** Responses related to participants’ intention to adhere to preventive measures.

(*N* = 746)	Responses	*n*	(*%*)
I will voluntarily adhere to the COVID-19 preventive measures recommended by the health authorities.	Strongly disagree	4	(0.5)
	Disagree	4	(0.5)
	Uncertain	27	(3.7)
	Agree	242	(32.4)
	Strongly agree	469	(62.9)

### 3.4 Regression analysis of expected increases in COVID-19 cases during the omicron wave

Table 3 presents the results of the univariate logistic regression analysis that examined the factors that predict the following dependent variable: the expectation of increased COVID-19 cases. The results showed that younger respondents were significantly more likely to expect an increase in COVID-19 cases (OR = 0.98; 95% CI: 0.96–0.99). Married respondents were 31% less likely to expect COVID-19 cases to increase. In addition, respondents with university degrees or higher were significantly more likely (1.54 times) to expect an increase in COVID-19 cases compared to those with pre-university educations. Finally, individuals who were more anxious about the Omicron variant (OR = 1.22; 95% CI: 1.15–1.30) or who were more worried about the reinstatement of lockdowns (OR = 1.10; 95% CI: 1.01–1.12) were more likely to expect an increase in COVID-19 cases.

**Table 3:** Univariate logistic regression for the different predictors of the expectation of increased COVID-19 cases during the Omicron wave (*N* = 746).

Variables		Perception of the anticipated increase vs. decrease in COVID-19 cases			
		B (SE)	OR (95% CI)	Wald	*p*-value
Age		−0.02 (0.01)	0.98 (0.96–0.99)	5.13	0.024*
Gender	Female		1		
	Male	−0.50 (0.16)	0.95 (0.69–1.31)	0.097	0.756
Marital status	Unmarried		1		
	Married	−0.37 (0.16)	0.69 (0.50–0.96)	4.96	0.026*
Care for dependents	No		1		
	Yes	−0.04 (0.16)	0.95 (0.69–1.31)	0.08	0.767
Education level	Pre-university		1		
	University or higher	0.43 (0.17)	1.54 (1.10–2.17)	6.26	0.012*
Contracted COVID-19	No		1		
	Yes	0.23 (0.16)	1.25 (0.91–1.73)	1.91	0.17
Adherence to prevention measures		−0.18 (0.12)	0.83 (0.66–1.06)	2.29	0.130
Anxiety about COVID-19		0.20 (0.03)	1.22 (1.15–1.30)	36.82	< 0.001*
Worry about the reinstatement of lockdowns		0.06 (0.03)	1.10 (1.01–1.12)	5.03	0.025*

B = coefficient for the constant, SE = standard error, OR = crude odds ratio, CI = 95% confidence interval, Wald = Wald chi-square test, 1 = the reference category for the variable, * = statistically significant at *p*-value < 0.05.

A multivariate logistic regression model was also implemented to help predict respondents’ expectations of increased COVID-19 cases (Table 4). The model included five factors that showed a significant association with the dependent variable at a *p*-value <0.05 in the univariate logistics regression model: age, marital status, education level, anxiety about COVID-19, and worry about the reinstatement of lockdowns. The model demonstrated a non-significant *p*-value (*p* > 0.05) for the Hosmer–Lemeshow goodness-of-fit test statistic, indicating that the data was well-fit to the model. The results showed that two of the five factors in this multivariate model were significant predictors of the respondents’ expectations (*p* < 0.05). The odds ratios indicated that anxiety about COVID-19 (AOR = 1.23; 95% CI: 1.15–1.32) was the strongest predictor of respondents’ expectation of increasing COVID-19 cases, followed by education level (AOR = 1.58; 95% CI: 1.11–2.25).

**Table 4:** Multivariate logistic regression for the different predictors of the expectation of increased COVID-19 cases during the Omicron wave (*N* = 746).

Variables		Perception of the anticipated increase vs. decrease in COVID-19 cases			
		B (SE)	AOR (95%CI)	Wald	*p*-value
Age		−0.01 (0.01)	0.99 (0.96–1.02)	0.60	0.438
Marital status	Unmarried		1		
	Married	−0.17 (0.25)	0.84 (0.52–1.37)	0.47	0.493
Education level	Pre-university		1		
	University or higher	0.46 (0.18)	1.58 (1.11–2.25)	6.49	0.011*
Anxiety about COVID-19		0.21 (0.04)	1.23 (1.15–1.32)	32.82	< 0.001*
Worry about the reinstatement of lockdowns		−0.01 (0.03)	0.99 (0.94–1.05)	0.099	0.754

B = coefficient for the constant, SE = standard error, AOR = adjusted crude odds ratio, CI = 95% confidence interval, Wald = Wald chi-square test, 1 = the reference category for the variable, * = statistically significant at *p*-value < 0.05.

### 3.5 Anxiety related to COVID-19 during the omicron wave and worries about the reinstatement of lockdowns

**Figure 3:**
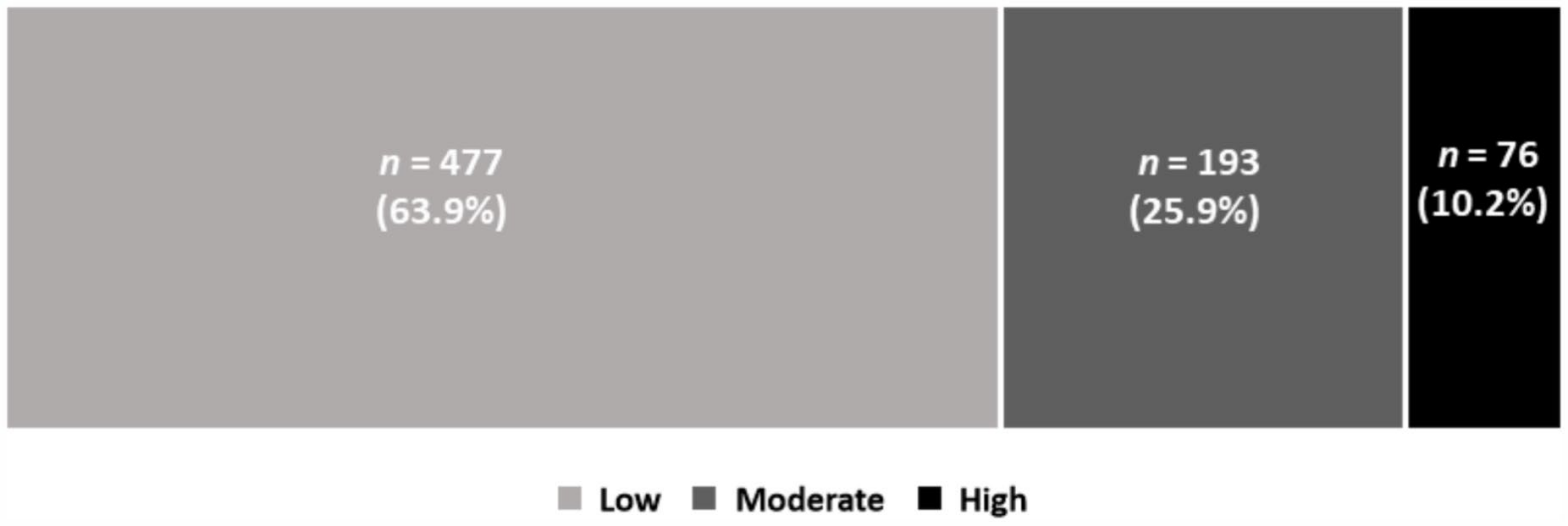
Anxiety levels related to COVID-19 during the Omicron wave.

The mean anxiety score was 3.11, with a standard deviation of 2.40. About 36.1% of the participants reported moderate to high anxiety levels. Figure 3 shows the distribution of low, moderate, and high anxiety levels related to COVID-19 among participants during the Omicron wave.

**Figure 4:**
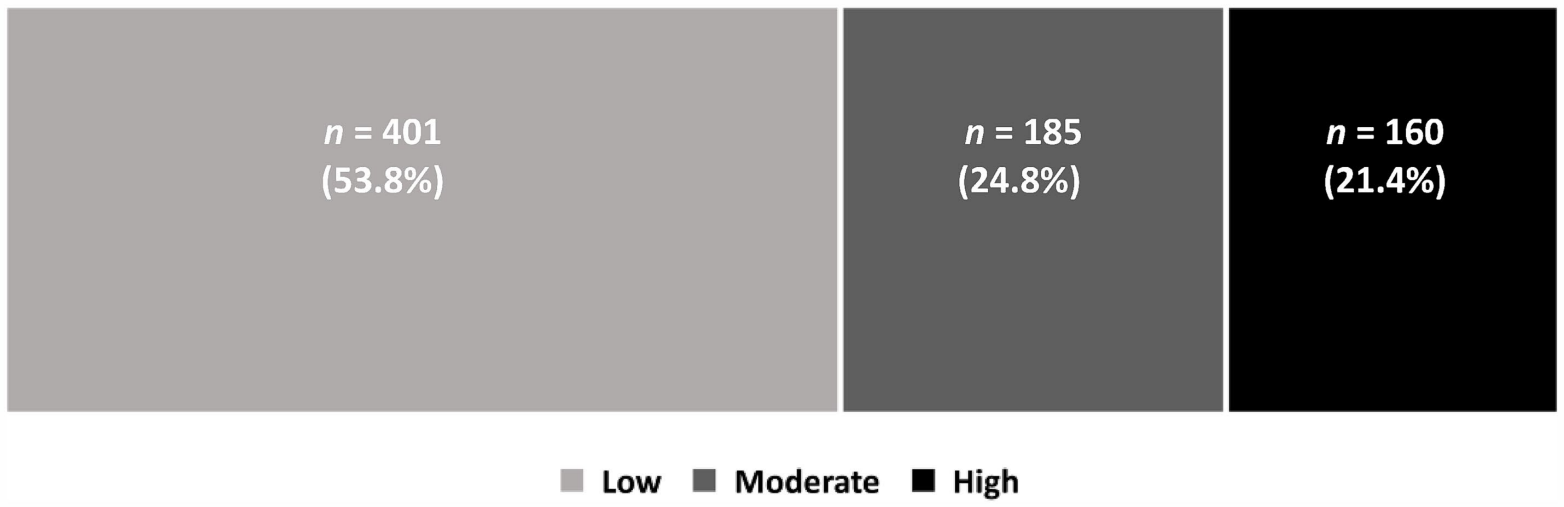
Worry levels related to the reinstatement of lockdown during the Omicron wave.

The mean score associated with the respondents’ worry about the reinstatement of lockdown was 3.84, with a standard deviation of 3.02. About 46.2% of the participants reported moderate to high worry levels. Figure 4 shows the distribution of low, moderate, and high worry levels regarding the reinstatement of lockdowns.

Ordinal logistic regression analysis was performed, with anxiety levels (low–moderate–high) as the dependent variable and the participants’ characteristics as the predictor variables. The model was fitted to the data (*p* < 0.05) and exhibited a 7% improvement in the prediction of anxiety levels compared to the null model. However, none of the factors exhibited a significant association with anxiety levels except for worries related to the reinstatement of lockdowns. Table 5 shows that the respondents who were more worried about the reinstatement of lockdowns were 1.28 times more likely to have a high level of anxiety related to COVID-19.

**Table 5:** Predictors of anxiety level using ordinal logistic regression analysis.

Variables		Anxiety level			
		B (SE)	AOR (95%CI)	Wald	*p*-value
Age		0.01 (0.01)	1.01 (0.99–1.04)	0.57	0.452
Gender	Female	0.16 (0.16)	1.17 (0.86–1.61)	0.98	0.322
	Male		1		
Marital status	Unmarried	0.32 (0.24)	1.73 (0.85–2.21)	1.70	0.193
	Married		1		
Care for dependents	No	−0.09 (0.18)	0.92 (0.64–1.31)	0.228	0.633
	Yes		1		
Education level	Pre-university	0.12 (0.16)	1.12 (0.81–1.55)	0.49	0.483
	University or higher		1		
Contracted COVID-19	No	−0.14 (0.16)	0.87 (0.63–1.20)	0.72	0.398
	Yes		1		
Adherence to prevention measures		−0.13 (0.12)	0.88 (0.69–1.12)	1.12	0.290
Worry about the reinstatement of lockdowns		0.25 (0.03)	1.28 (1.21–1.35)	84.41	< 0.001*

B = coefficient for the constant, SE = standard error, AOR = adjusted crude odds ratio, CI = 95% confidence interval, Wald = Wald chi-square test, 1 = the reference category for the variable, * = statistically significant at *p*-value < 0.05.

## 4 Discussion

Understanding how people perceive and respond to pandemics is crucial for effective management. The COVID-19 pandemic has posed a significant threat to health over the past few years and has affected many aspects of daily life. In particular, the rapid spread of the Omicron variant led to a substantial increase in COVID-19 cases across many countries worldwide, including Saudi Arabia (25–30). Despite this, our study showed differences in people’s expectations regarding future COVID-19 trends. Over one-third of the participants thought that COVID-19 cases would increase, 27.3% believed that cases would decrease, and the remaining 42.5% were uncertain. Public concerns regarding the increasing number of COVID-19 cases caused by the Omicron variant in Saudi Arabia have been documented in research studies (26) and local media (31). How people perceive a disease’s severity and contagiousness will significantly impact their response behaviors to reduce susceptibility (32). When people perceive a threat to their health and believe that the benefits of a preventive behavior outweigh its potential downsides, they are more likely to adopt that behavior (33). Participants in this study identified several factors that influenced their expectation that COVID-19 cases were likely to increase, including people’s non-compliance with preventive measures and the virus’s high transmissibility.

Prior research has shown that non-compliance with COVID-19 preventive measures (34) and the rapid spread of the virus (35) increased the risk of both contracting and transmitting the disease. Reluctance to follow the recommended preventive measures was observed in many regions worldwide (36–40), including Saudi Arabia (41). Unexpectedly, our study revealed that a few participants were unwilling to adhere to preventive measures against COVID-19 because they believed that the virus was not real or risky or because they had already been vaccinated. Nearly 28% of the participants expected the spread of COVID-19 to be limited during the Omicron wave because they thought the virus was less transmissible than previous variants. This suggests that there is a concerning portion of the population that does not take COVID-19 seriously. Similar attitudes have been observed worldwide, with some people refusing to acknowledge the reality of COVID-19 (40, 42–44). Participants who did not believe in the existence of the virus had reduced perceptions of risk and did not adhere to risk-prevention behaviors (44). Constantinou, Kagialis and Karekla (42) found that as the strength of belief in conspiracy theories increases, the trust in COVID-19 science and willingness to adhere to preventive measures decreases significantly. This is consistent with a recent study from Spain, where young people who believed in conspiracy theories were less likely to follow COVID-19 preventive measures (39). Furthermore, a large-scale study found a decrease in people’s anxiety levels following the emergence of the Omicron variant (15), suggesting that prolonged exposure to health risks may result in reduced levels of anxiety in some individuals. Some studies suggest that ongoing uncertainties linked to COVID-19 may result in “worry fatigue,” reducing the public’s desire to adhere to preventive measures (45). Previous research, including a study conducted in Saudi Arabia, have shown that anxiety and depressive symptoms tend to decrease after receiving the COVID-19 vaccine (46–48). Consequently, continuous efforts are essential to strengthening and enforcing preventive measures and policies aimed at controlling virus transmission during pandemics.

Participants of this study who believed that COVID-19 cases were likely to decrease cited factors such as compliance with preventive measures, accessibility of free vaccines, health education programs, and government policies and interventions. Many people were initially hesitant to get vaccinated against COVID-19, but the distribution of free vaccines in several countries, including Saudi Arabia, motivated individuals to prioritize their safety as well as that of their families, extending even to the broader community (49). Previous research has shown that people are more likely to adhere to preventive measures if they believe that the government and healthcare system are taking effective actions to control the spread of disease (50, 51). Conversely, mistrust in the government-led responses reduces adherence to COVID-19 preventive measures (40).

Our findings revealed that there was a significant association between the respondents’ expectation of increased COVID-19 cases and both their education levels and their anxiety about the Omicron variant. Individuals with higher anxiety levels were more likely to expect a rise in COVID-19 cases than those with fewer concerns. Similarly, individuals who possessed university-level education or higher were 1.58 times more likely to expect a rise in COVID-19 cases compared to those who had only attained a pre-university qualification. These findings suggest that perceptions and expectations of the pandemic vary according to demographic factors such as educational background and general anxiety levels.

Anxiety is a common issue for many people during pandemics and health crises. According to the WHO, there was a 25% increase in the worldwide occurrence of anxiety and depression during the first year of the COVID-19 pandemic (52). Increased anxiety can have negative impacts on an individual’s overall health, their perspective on emerging health threats, and the extent to which they adhere to preventive measures. Several studies conducted in Saudi Arabia on the degree of anxiety experienced by individuals due to COVID-19 reported inconsistent results: some reported high or moderate levels of anxiety among participants (53–55) while others reported lower levels of anxiety (56, 57). A sentiment analysis of global tweets related to the Omicron variant revealed a high level of worry about the emergence of this strain (58). However, sentiment in Saudi Arabia was relatively neutral, with no significant concerns being observed (58). Nevertheless, over one-third of our study respondents exhibited moderate to high anxiety levels relating to COVID-19 and the Omicron variant. Although the majority of participants were already familiar with COVID-19, some may have felt more uncertain about the new variant. Concerns about the reinstatement of lockdowns also significantly heightened anxiety levels related to COVID-19. However, the other examined demographic variables were found to be insignificant. These findings contrast with previous studies from Saudi Arabia, which found that personal factors, including age (57, 59, 60), gender (53, 59–61), level of education (60), and past infection with SARS-CoV-2 (61) were potential influencers of COVID-19-related anxiety.

In response to the COVID-19 pandemic, many countries, including Saudi Arabia, implemented lockdowns as a preventive measure (62). These lockdowns imposed various restrictions, such as isolating sick individuals, stay-at-home orders, curfews, and limits on commercial and service activities. While effective in limiting the spread of the disease, the lockdowns significantly disrupted various aspects of daily life and contributed to psychological stress for many individuals (63–65). Approximately 46% of respondents in this study expressed moderate to high levels of concern about the possibility of a new lockdown. The results from previous research have revealed the negative role of lockdowns on people’s mental health (66–69). Experts in mental health have warned that prolonged lockdowns, quarantine, and isolation could cause mental health issues and increased suicide rates due to physical inactivity, economic hardship, and social isolation (70).

Our study contributes to COVID-19 research in Saudi Arabia by shedding light on how the Saudi public viewed COVID-19 during the Omicron wave—one of the most prevalent and infectious variants. Understanding how people’s perceptions evolve during pandemics is crucial for decision-makers in order to improve preventive plans and allocate resources effectively. However, our study has certain limitations. The main limitation is the use of a non-probability sampling technique due to COVID-19 constraints. In addition, the survey was conducted online, which limited its scope to educated people and those with Internet access. These limitations must be considered when interpreting the findings, as they may affect the generalizability of the results.

## 5 Recommendations for policy improvement

The findings of our study provide valuable insights into Saudi Arabia’s response to the COVID-19 pandemic as well as public perception and policy development. Saudi Arabia implemented several policies, including containment and closure policies, health system measures, vaccination, and economic policies (71). Containment and closure policies refer to the administrative measures used to limit people’s mobility, such as closing workplaces and restricting travel, to reduce transmission within the population. Health system policies refer to public health measures such as testing, contact tracing, delivering vaccines, wearing facial masks, protecting older people, and strengthening the health system. Vaccination policies include plans that provide free vaccinations to the population. Priority was given to individuals most at risk of developing severe symptoms, such as senior citizens and people with chronic diseases, and those most at risk of infection, such as healthcare workers. In addition, people participating in indoor activities were required to receive vaccination. Economic policies refer to the policies implemented by the Saudi government to address the financial impact of COVID-19. These policies included financial support for job seekers, deferred financial obligations for households, incentives for economic spending, and financial aid to other nations (71, 72).

COVID-19 mitigation policies in Saudi Arabia varied throughout the pandemic. In the early waves of the pandemic, measures such as strict lockdowns and mandatory testing were implemented, but as the pandemic continued, the focus shifted toward localized restrictions and expanded vaccination efforts (73, 74). Despite the Saudi government’s outstanding response to COVID-19, targeted improvements to specific zones could strengthen future preparedness.

Our study emphasized the importance of customizing communication strategies for different groups during pandemics. Educational background and anxiety levels played a significant role in influencing people’s expectations about the trend in COVID-19 cases. There was also a high level of concern about the possibility of future lockdowns. It seems that the strict measures enforced during the early waves of the pandemic had a significant psychological impact that may have had lasting adverse effects on the health of Saudi citizens (70, 75). The development of customized policies that target disadvantaged groups, address emerging challenges, and build trust in public health initiatives is essential to better manage future epidemiological outbreaks and pandemics.

Interestingly, a small percentage of participants in our study still doubted the existence and severity of the virus, highlighting gaps in communication and awareness efforts. Future public health responses could address this issue through prompt, targeted campaigns in response to specific concerns and misinformation. The public’s positive perception of government measures, reflected in the expectation of declining cases, highlights the crucial role of effective leadership during a pandemic.

These points highlight the importance of investing in future pandemic preparedness as well as building long-term resilience. Establishing effective surveillance systems and early-warning mechanisms is essential for proactive policy development. Additionally, ensuring clear and timely communication about new variants can help prevent panic and reduce the spread of misinformation, especially during the early stages of the pandemic.

Overall, Saudi Arabia can reinforce its public health framework by drawing lessons from past achievements and challenges. Key areas for improvement include targeted communication initiatives, comprehensive preparedness plans, and effective early-alarm systems—all crucial to effectively handling future health crises.

## 6 Conclusion

Our findings revealed that most participants were uncertain about the trend of Omicron infections. Individuals with higher levels of anxiety and those with higher education levels were more likely to expect an increase in COVID-19 cases. Individuals who were worried about the potential of further lockdowns or a rise in COVID-19 cases also exhibited higher levels of anxiety. The possibility of another lockdown caused moderate to high concern in less than half of the participants. Additionally, a small number of participants refused to follow preventive measures, either believing that the virus was not real or that it did not pose a significant risk. Some participants also believed they were fully protected after receiving three doses of the vaccine. Such misconceptions are concerning as they lead individuals to underestimate the risks associated with COVID-19 and future pandemics.

This study provides insights into how Saudi citizens perceived the Omicron wave of the COVID-19 pandemic and the factors that shaped their expectations of the disease trends. Given that COVID-19 is likely to continue to be a concern, we may face additional waves of infectious and potentially risky variants in the future. Thus, it would be interesting to explore how people’s perceptions change as the virus evolves using a larger and more representative sample. It would also be helpful to assess the impact of different cultural factors on people’s perceptions of SARS-CoV-2 variants. Finally, future studies should aim to include underrepresented groups, such as older people, illiterate individuals, immigrants, and those without Internet access.

## Data Availability

The raw data supporting the conclusions of this article will be made available by the author without undue reservation.
