# Supplementary material for: Public perception of COVID-19 in Saudi Arabia during the Omicron wave: recommendations for policy improvement
Source: Front Public Health. 2025 Feb 27;13:1419891. doi: 10.3389/fpubh.2025.1419891 (PMC11903483; doi:10.3389/fpubh.2025.1419891)
Supplement: Supplementary file 1 [file Data_Sheet_1.PDF]

Public perception of COVID-19 in Saudi Arabia during the Omicron variant

Supplementary Material

Supplementary Materials: Questionnaire

Note: Researchers may use and modify this questionnaire without prior permission as long as they cite the source.

COVID-19 Omicron is a coronavirus variant identified for the first time in South Africa in November 2021.

Dear citizens and residents of Saudi Arabia,

This survey aims to study Saudi citizens' perception of COVID-19 during the Omicron variant. We look forward to your participation and support. Taking part in this study is voluntary anonymous. Data from this tool will be used for research purposes only according to the requirements of the Research Ethics Committee. Answering all questions will enable us to analyze this questionnaire's data and obtain accurate findings. In addition, your participation will support the national and international scientific efforts to confront the pandemic. Finally, filling out the questionnaire takes only about three minutes.

For Further Information, you may contact Mohammed
Almalki
College of Public Health and Tropical Medicine
Jazan University mjalmalki@jazanu.edu.sa

Thank you.

a Are you a Saudi citizen and living in Saudi Arabia?

- ☐ Yes (please go to the next question)
- ☐ No (you may stop here, thank you)

b I am 18 years or older, understand the aim of this research, and agree to participate voluntarily.

- ☐ Yes (please start the questionnaire)
- ☐ No (you may stop here, thank you)

Part one: Participant Demographics

1. Gender

   - Male
   - Female

2. Age ______________________________

3. Marital status

   - Married
   - Non-married

4. I live with my family in the same house.

   - Yes
   - No

5. I personally take care of dependents such as children, older people, or patients

   - Yes
   - No

6. Education level

   - Illiterate (the questionnaire was filled out by someone else)
   - Primary
   - Intermediate
   - Secondary or equivalent
   - Post-secondary diploma
   - Bachelor's degree
   - Higher education

7. Have you contracted COVID-19?

   - Yes
   - No

8. Province you live in:

- Riyadh Province
- Makkah Province
- Madinah Province
- Qassim Province
- Eastern Province
- Aseer Province
- Tabuk Province
- Ha'il Province
- Northern Borders Province
- Jazan Province
- Najran Province
- Al-Bahah Province
- Al-Jawf Province

9. City or governorate you live in: ______________________________

Part two: Response to the COVID-19 Omicron Variant

10. On a scale of 1 to 10 (the higher the scores, the higher the anxiety level), how anxious are you about the Omicron variant compared to when COVID-19 first emerged?

	1	2	3	4	5	6	7	8	9	10	
No anxiety	○	○	○	○	○	○	○	○	○	○	High anxiety

11. I expect COVID-19 cases to increase in Saudi Arabia during the Omicron variant.

- Strongly Agree
- Agree
- Uncertain
- Disagree
- Strongly Disagree

12. If you expect COVID-19 cases to increase during Omicron, what are the possible reasons for that?

Check all that apply.

- [ ] Lack of people's adherence to the preventive measures
- [ ] Limited health awareness activities
- [ ] Limited government measures to confront the pandemic
- [ ] Limited availability of COVID-19 vaccines
- [ ] High transmissibility of Omicron variant
- [ ] Other: ________________________________

13. If you expect COVID-19 cases to decrease during Omicron, what are the possible reasons for that?

Check all that apply.

- [ ] People's adherence to the preventive measures
- [ ] Health awareness activities
- [ ] Government measures to confront the pandemic
- [ ] Availability of free COVID-19 vaccines for all
- [ ] Low transmissibility of Omicron variant
- [ ] Other: ________________________________

14. On a scale of 1 to 10 (the higher the scores, the higher the level of worry), how worried are you about the possibility of another lockdown to control the Omicron infection?

	1	2	3	4	5	6	7	8	9	10	
No worry	○	○	○	○	○	○	○	○	○	○	High worry

15. I will voluntarily adhere to the preventive measures approved by the health and government authorities.

- ○ Strongly Agree
- ○ Agree
- ○ Uncertain
- ○ Disagree
- ○ Strongly Disagree

16. Any additional information to enhance the results of the study?
